# Upgraded bidirectional approach video-assisted neck surgery (BAVANS) using a rigid endoscope with variable viewing direction for advanced endoscopic lymph node dissection in thyroid cancer patients

**DOI:** 10.1007/s00595-019-01909-3

**Published:** 2019-11-05

**Authors:** Akihiro Nakajo, Koji Minami, Yoshiaki Shinden, Hiroko Toda, Tadahiro Hirashima, Ayako Nagata, Yuki Nomoto, Kosei Maemura, Shoji Natsugoe

**Affiliations:** Department of Surgical Oncology, Digestive Surgery, Breast and Thyroid Surgery, Kagoshima University, 8-35-1 Sakuragaoka, Kagoshima City, Kagoshima, 890-0075 Japan

**Keywords:** BAVANS, Video-assisted neck surgery, Lymph node dissection

## Abstract

In 2011, we developed bidirectional approach video-assisted neck surgery (BAVANS) for endoscopic thyroid cancer surgery. BAVANS combines two different approach pathways at 180 degrees to the cervical lesion for endoscopic thyroidectomy and complete cervical lymphadenectomy. We reported previously that the cranio-caudal approach is extremely useful for endoscopic complete lymph node dissection around the trachea. In 2014, we upgraded the initial BAVANS for better maneuverability and quality of lymph node dissection. A new high-tech rigid endoscope with a variable viewing direction (EndoCAMeleon™), has enabled us to reduce the camera port in the anterior neck while keeping the easy maneuverability and the same quality of central lymph node dissection (LND) as with the initial BAVANS. Endoscopic thyroid cancer surgery is now evolving concurrently with new visual technology.

## Introduction

**Fig. 1:**
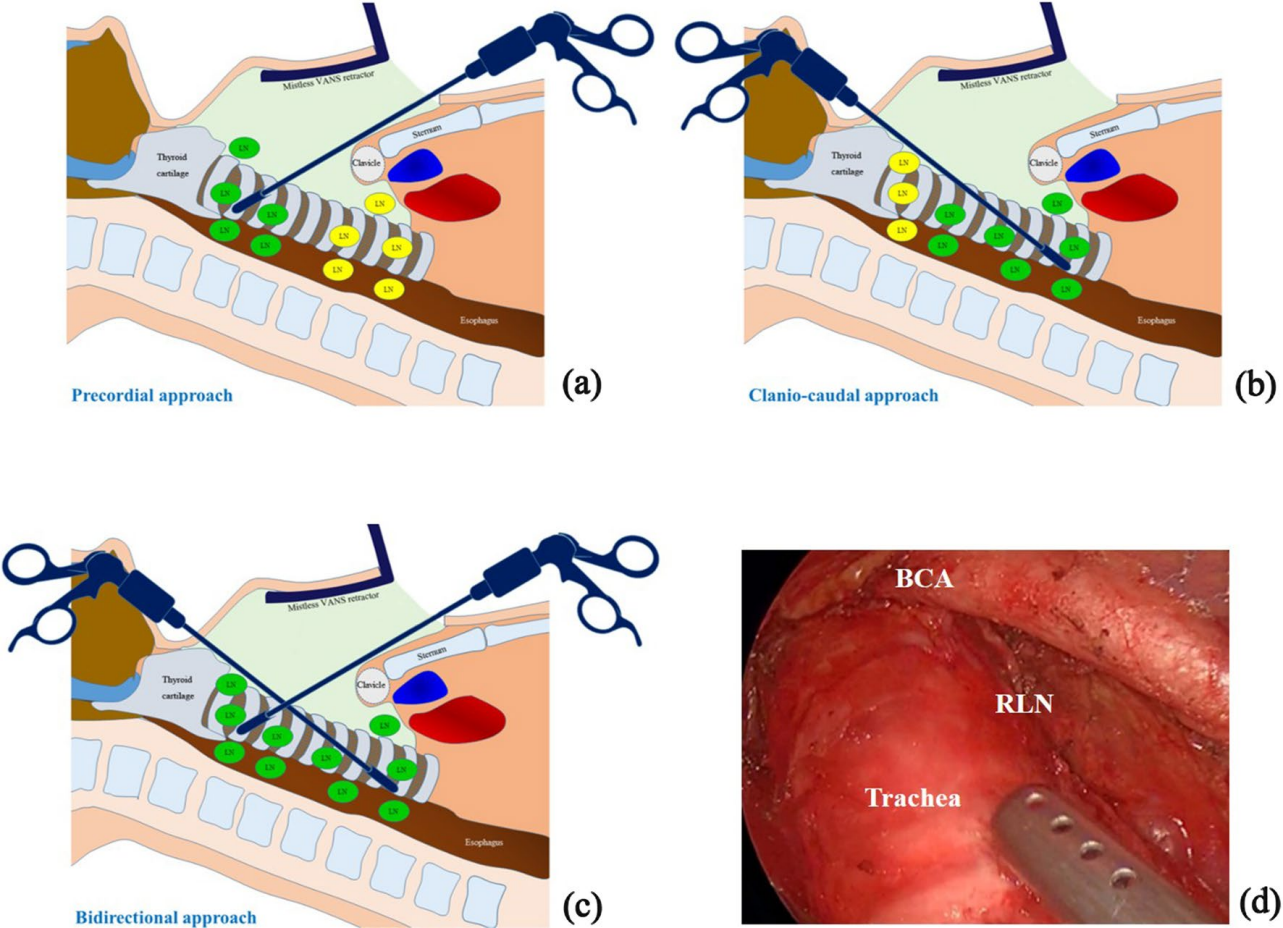
**a** Precordial approach. With the transaxillary or precordial approach, the clavicle or sternum obstructs lymph node dissection in the area just behind the sternal notch or clavicle, shown here as yellow nodes. **b** Craniocaudal approach. Under the submandibular craniocaudal approach, access to the middle and lower paratracheal lymph nodes is simple. **c** Bidirectional approach. Complete lymph node dissection can be achieved easily with this combined approach. **d** Lymph node dissection of the right side via the craniocaudal approach. The recurrent laryngeal nerve is clearly identified. *RLN* recurrent laryngeal nerve; *BCA* brachiocephalic artery

Various techniques of endoscopic and robotic thyroid surgery have been reported recently; especially those featuring extracervical approaches, including precordial, transaxillary, transareola, facelift, or transoral approaches, alone or in combination [1–21]. These approaches have great cosmetic advantage and have become standard in high volume centers. In endoscopic thyroid cancer surgery, the quality of lymph node dissection (LND) is important because some cases of papillary thyroid cancer require complete LND, especially around the trachea. With the aim of establishing an endoscopic surgical method for complete cervical LND, we developed bidirectional approach video-assisted neck surgery (BAVANS) in 2011 and reported subsequently that the cranio-caudal approach is extremely useful for endoscopic complete LND around the trachea [22] (Fig. 1). In 2014, we upgraded this initial BAVANS for better maneuverability and quality of LND by introducing a new rigid endoscope featuring variable viewing direction (EndoCAMeleon™).

## Methods

**Fig. 2:**
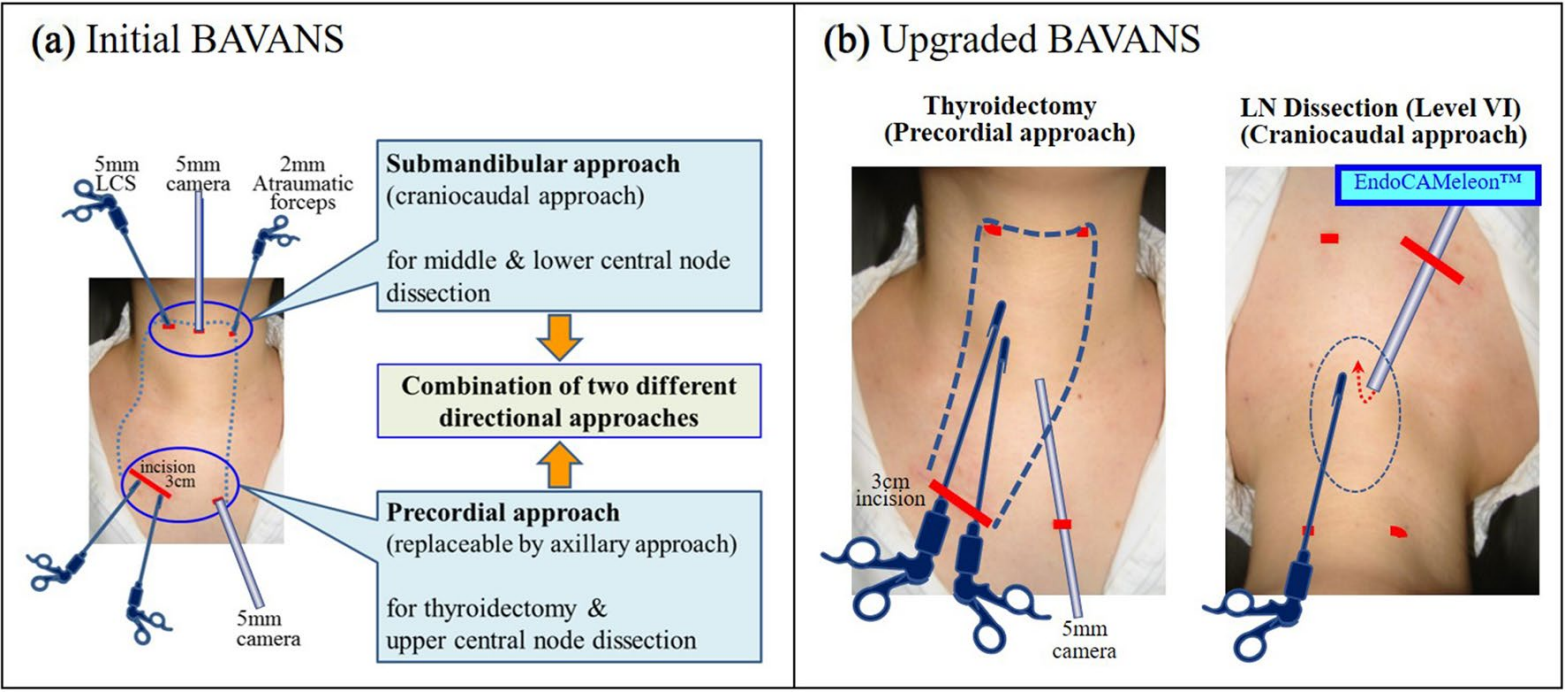
**a** The initial BAVANS required three ports in the visible upper neck area to obtain the cranio-caudal view. We inserted the 5 mm oblique rigid endoscope from the midline point of the submandibular area in the caudal direction. **b** The EndoCAMeleon™ allows us to solve the two problems associated with initial BAVANS, achieving easy maneuverability during the craniocaudal approach, as well as the cosmetic benefit of elimination of the midline port on the neck with its noticeable port scar

BAVANS is a combination procedure involving two-directional approaches at 180°: the precordial approach (or axillary approach) for the first half of surgery to perform thyroidectomy and dissection of the upper part of the central node (upper level VI), and the submandibular approach for the second half of the surgery for middle and lower central node lymphadenectomy (middle and lower level VI). The surgeon changes the location where they stand for each approach (Fig. 2).

### Body position and setting of the equipment

The patient is placed in the supine position with slight neck extension achieved by a shoulder pillow, under general endotracheal anesthesia. Because the surgeon must be positioned over the head of the patient and have access to the central node from the submandibular neck area for the second half of surgery, the anesthetic equipment is set up to the right of the patient.

## Upgraded BAVANS procedure

### Thyroidectomy

First, total or hemithyroidectomy is performed, followed by LND of the upper part of the central node via a gasless precordial (or axillary) approach using a 5 mm oblique tip endoscope with a 30° angle. When performing thyroidectomy, the axillary approach or areolar approach is possible, but we generally select a precordial approach with modification of the video-assisted neck surgery (VANS) technique developed by Shimizu et al. [3]. In the precordial approach, a 3-cm skin incision is made in the right anterior chest area, 6 cm beneath the inferior margin of the clavicle, as well as a 5-mm camera port incision in the left side. (Fig.  2b). Dissection is performed along with the subplatysmal layer, extending up to the level of the neck wrinkle above the thyroid cartilage. Laterally, this dissection can be continued up to the medial border of the sternocleidomastoid muscle on the tumor side, and up to the contralateral internal borderline of the sternocleidomastoid muscle in patients undergoing lobectomy. In total thyroidectomy, lateral dissection is extended up to the medial borderline of the sternocleidomastoid muscles on both sides. After dissection of the subplatysmal plane, a mistless VANS retractor is inserted through the right main incision and elevated by the retracting wire system fixed to the pole above the patient’s neck. Pulling the strap muscles laterally with an exclusive detachable wire retractor creates an excellent visual field and working space. Following dissection around the thyroid gland, all the vessels, including the upper and lower thyroid arteries and veins, are transected by using ultrasonic coagulating shears. The recurrent laryngeal nerve and outer branch of the superior laryngeal nerve are identified and isolated by using a nerve integrity monitoring system (NIM-Response^®^ 3.0). More details are provided in our previous article describing BAVANS [22].

### Dissection of the upper part of the central node

After thyroidectomy, LND of the upper part of the central nodes (upper area in level VI) is performed by extending the precordial approach. Here, the upper part of the central node refers to the lymph node located above the horizontal line, 2 cm beneath the inferior border of the cricoid cartilage.

### Dissection of the lower part of the central node

**Fig. 3:**
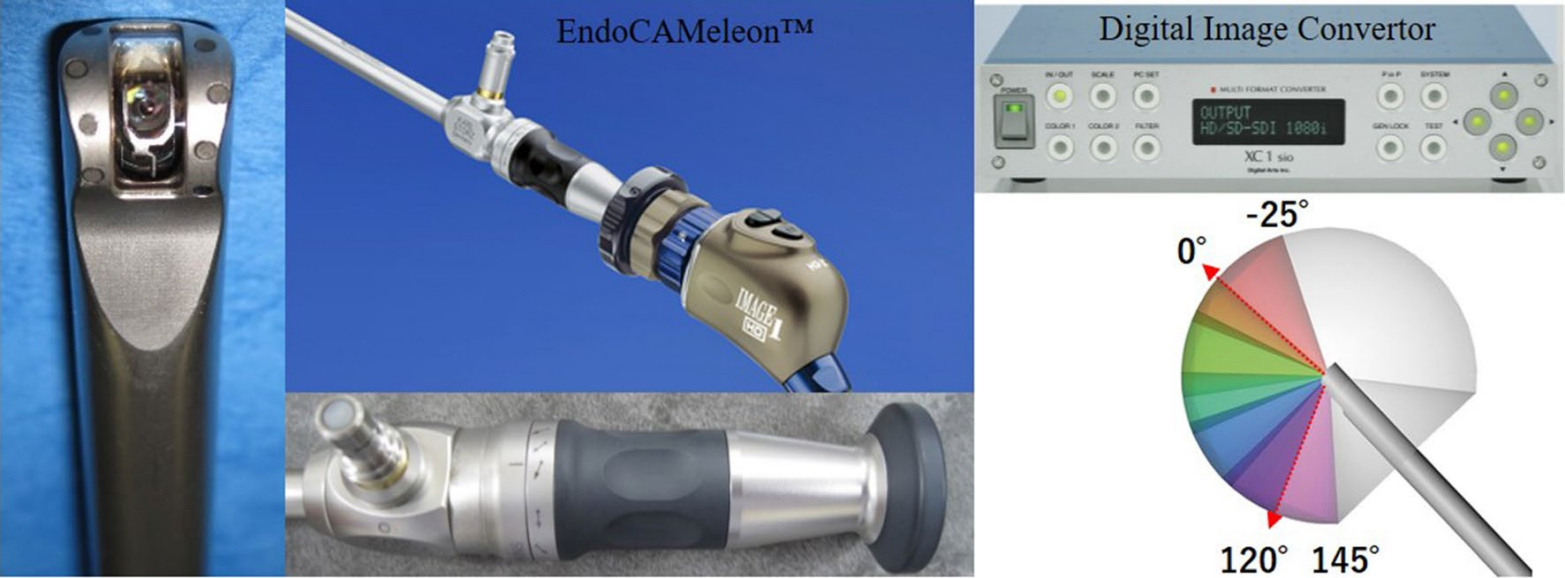
The EndoCAMeleon™ (Karl Storz SE & Co. KG, Tuttlingen, Germany), a 10 mm rigid endoscope with a variable direction of view of 0°–120°. This specific telescope allows us to see the reverse image around the lower part of the trachea by turning the knob. Although this reverse image created by the EndoCAMeleon™ requires image correction of the vertical and horizontal direction, it can be corrected instantly by using the digital image converter

After dissection of the upper part of the central node via the precordial approach, we change from a 5 mm 30° oblique rigid endoscope to a 10 mm rigid endoscope with a variable direction of view of 0°–120°. This endoscope is inserted from the main incision on the right chest wall in a cranial direction, holding it in place with a flexible telescope holder (Lockarm^®^, System JP, Hamamatsu, Japan). This specific telescope, called an EndoCAMeleon™ (Karl Storz SE & Co. KG, Tuttlingen, Germany), can be used to obtain a reverse image around the lower part of the trachea by turning the knob on the endoscope (Fig. 3). After adjustment, the surgeon moves to a position over the head of the patient to approach the lower paratracheal area from the cranial side. Two exclusive ports, 3.5 mm and 5 mm, are inserted into the submandibular area of the anterior neck. The 5-mm port in the right cervical lesion is used for dissecting forceps, a suction tube, and ultrasonic shears or endoscopic scissors, and the 3.5-mm left port is used for the atraumatic gripping forceps. The surgeon can perform delicate and meticulous dissection while preserving the recurrent laryngeal nerve with the aid of a clear, magnified endoscopic craniocaudal view created by the EndoCAMeleon™ inserted from the opposite side (Fig. 2b). Although this reverse image obtained by the EndoCAMeleon™ requires mirror image correction, this can be done using a digital image converter (Fig. 3). After complete LND, a drainage tube is placed beside the trachea.

## Results

The average operating times for the upgraded BAVANS were 193 min and 245 min in the hemithyroidectomy with unilateral central node dissection (CND) group and the total thyroidectomy with bilateral CND group, respectively. The mean blood loss with the upgraded BAVANS was 26 ± 16.4 ml (*n* = 5) for total thyroidectomy with bilateral CND and 15.6 ± 26.8 ml (*n* = 46) for hemithyroidectomy with unilateral CND. The average number of harvested lymph nodes in unilateral and bilateral CND was 10 ± 4.5 (*n* = 8) and 7.4 ± 3.8 (*n* = 5), respectively, in the initial BAVANS vs. 9.2 ± 6.1 (*n* = 46) and 15.2 ± 8.5 (*n* = 6), respectively, in the upgraded BAVAS.

## Discussion

**Fig. 4:**
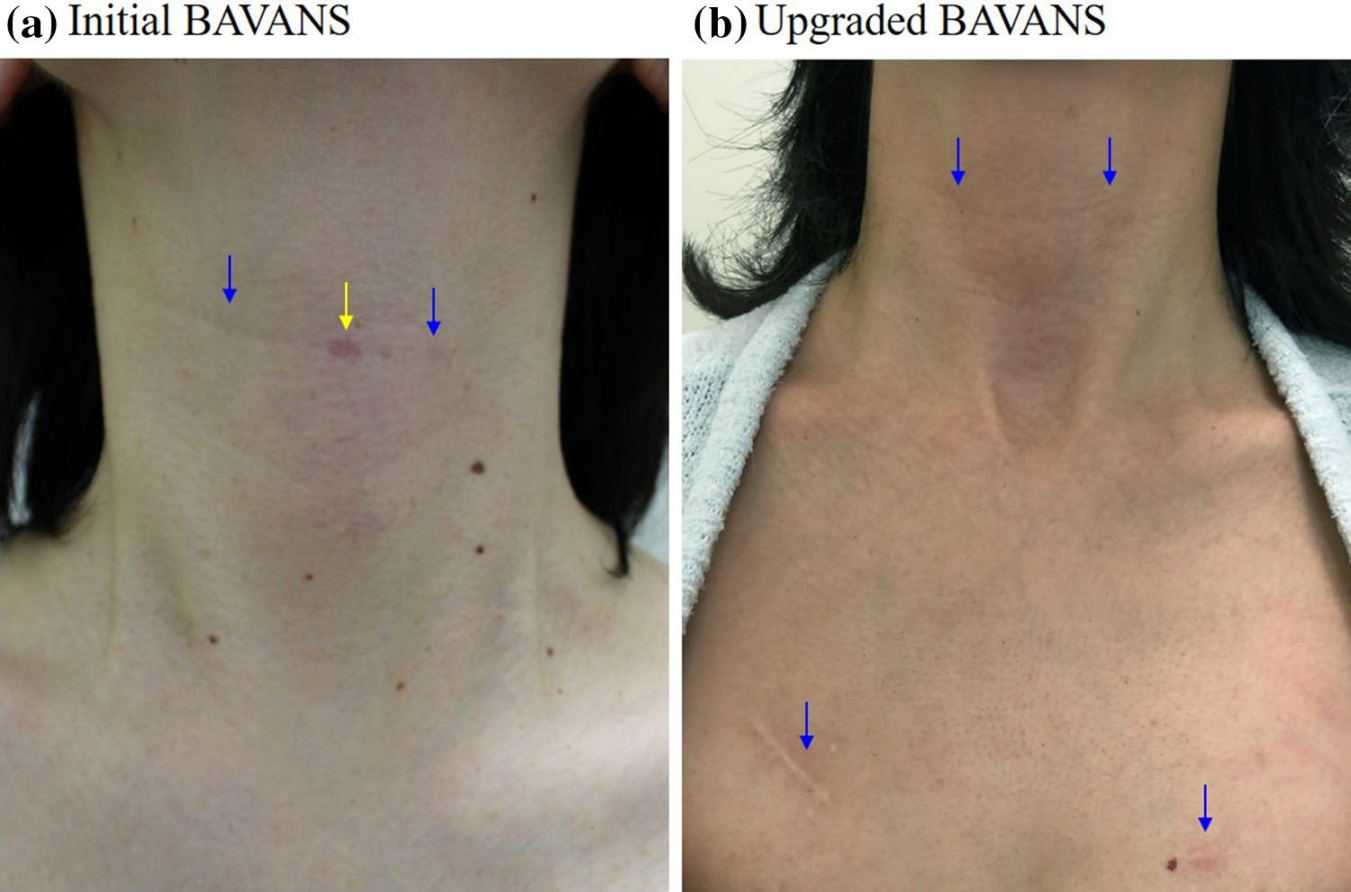
**a** The midline port scar tends to be more noticeable than the lateral port after the initial BAVANS. **b** There is no visible midline scar after the upgraded BAVANS. The scars on both sides in the submandibular area are entirely inconspicuous and the scars on the chest wall are hidden under the clothes

To perform complete cervical LND for papillary thyroid cancer, we developed BAVANS in 2011. We believe that the cranio-caudal approach is ideal for endoscopic complete LND. BAVANS allows for endoscopic lymphadenectomy to be performed under an excellent craniocaudal view, with safe endoscopic complete LND in patients with thyroid cancer. However, the initial BAVANS required the insertion of three ports in the visible upper neck area to obtain a cranio-caudal view, with a 5-mm oblique rigid endoscope inserted from the midline point on the submandibular area toward the caudal direction (Fig. 2a). However, our experience revealed two drawbacks. First, the midline port scar tends to be more noticeable than the lateral port; second, there is some limitation of the manipulation of the forceps because of the long endoscope occupying the space in front of the operator. Now, through the availability of a new rigid endoscope with variable direction of view, we can obtain not only the easy maneuverability of the craniocaudal approach but also the cosmetic benefit by elimination of the midline port on the neck. The EndoCAMeleon™ solves these two problems of the initial BAVANS, simultaneously (Fig. 4). The one-touch correction of the mirror image created by EndoCAMeleon™ inserted from the opposite side, made possible by the digital image converter, is spectacular. Our research demonstrates that the same or a greater number of lymph nodes can be extracted in BAVANS as in conventional open surgery. Furthermore, comparison of the initial BAVANS with the upgraded BAVANS shows that the number of harvested lymph nodes in the upgraded BAVANS is the same or greater. This suggests a curability advantage of the upgraded BAVANS in terms of oncological safety. Although the operative time for hemithyroidectomy with LND is about 1 h longer than that for conventional open surgery, BAVANS seems to surpass the open method in central node dissection.

## Conclusion

The combination approach of two different directions is a useful and promising surgical procedure for complete lymph node dissection in thyroid cancer surgery. In particular, the cranio-caudal approach is effective for lymphadenectomy of the lower paratracheal area and the supraclavicular area. The introduction of a new rigid endoscope featuring variable viewing direction improves the efficiency of BAVANS. If this rigid “Endowrist” camera is introduced into a medical robot system represented by DaVinci, its working range and validity will be further expanded. Endoscopic thyroid surgery involving robotics will advance with new developments in visual technology and surgical instruments.
